# Detection of Penicillium-Toxins in Nuts Commercialized in Italy Through LC-MS/MS Analyses

**DOI:** 10.3390/toxins18010012

**Published:** 2025-12-24

**Authors:** Fabio Buonsenso, Giovanna Roberta Meloni, Davide Spadaro

**Affiliations:** 1 Department of Agricultural, Forest and Food Sciences (DISAFA), University of Turin, Largo Paolo Braccini 2, 10095 Grugliasco, Italy; fabio.buonsenso@crea.gov.it (F.B.); giovannaroberta.meloni@gmail.com (G.R.M.); 2 Interdepartmental Centre for Innovation in the Agro-Environmental Sector—AGROINNOVA, University of Turin, Largo Paolo Braccini 2, 10095 Grugliasco, Italy; 3 Research Centre for Plant Protection and Certification—Council for Agricultural Research and Economics (CREA-DC), Via Carlo Giuseppe Bertero 22, 00156 Rome, Italy

**Keywords:** mycotoxins, mycotoxigenic fungi, food safety, postharvest pathogens

## Abstract

The consumption of nuts is widespread globally and constitutes a significant component of the human diet due to its nutritional value. However, the presence of mycotoxins in food products, including nuts, is a global public health concern. Mycotoxins are toxic metabolites produced by contaminating fungi such as *Aspergillus* spp. and *Penicillium* spp., which can contaminate crops during growth, harvesting, storage, or transport. The aim of this study was to conduct monitoring for the presence of mycotoxins in nuts already on the market. Specifically, secondary metabolites produced by *Penicillium* spp., including ochratoxin A, patulin, citrinin, cyclopiazonic acid, citreoviridin, griseofulvin, meleagrin, mycophenolic acid, penitrem A, roquefortine C, penicillins G and V, sulochrin, andrastin A, asterriquinone, chaetoglobosin A, cyclopenin, cyclopenol, and viridicatin, were investigated. Commercial products were purchased from various retail outlets in different formats, origins, and cultivation methods to assess potential influences of these factors on mycotoxin presence. Regarding Penicillium-toxins, 37% of the samples showed the presence of at least one of them, and 9% showed the simultaneous presence of two or more Penicillium-toxins. Peanuts had the highest incidence of Penicillium-toxin contamination, with at least one metabolite detected in 60% of the analyzed samples. The most common secondary metabolite among the samples was patulin (14%), while the secondary metabolite with the highest concentration was viridicatin in a walnut sample (151.40 ± 64.30 µg/kg). Besides Penicillium-toxins, aflatoxins were also analyzed with another validated LC-MS/MS method, but they were not detected in any sample. Although most Penicillium-toxins, and in particular patulin in nuts, are not currently regulated in the international legislation, they exert toxic effects on humans and animals, and their occurrence can represent a food safety risk.

## 1. Introduction

Contamination by filamentous fungi poses a significant challenge to the agricultural industry, particularly in production and commercialization of nuts, due to their potential to produce secondary metabolites toxic to humans and animals [1,2,3]. The primary mycotoxigenic fungi affecting nuts belong to the genera *Aspergillus* spp., *Penicillium* spp., *Fusarium* spp., *Claviceps* spp. and *Alternaria* spp. [4,5]. While *Fusarium* spp., *Claviceps* spp., and *Alternaria* spp. primarily infect crops during cultivation, *Aspergillus* spp. and *Penicillium* spp. are more commonly associated with postharvest contamination, although they can also infect crops in the field [6,7].

Fungal growth depends heavily on environmental factors such as temperature, water activity (a*_w_*), and substrate composition. Pre- and postharvest storage conditions, including temperature, humidity, and duration, directly influence fungal growth and mycotoxin production, and thus must be considered when interpreting measured contamination levels. For example, *Fusarium* spp. thrive in moist conditions with optimal a*_w_* levels of 0.99 but can tolerate levels as low as 0.85. Their ideal growth temperatures range between 20 °C and 25 °C. Conversely, *Aspergillus* spp. and *Penicillium* spp. can develop under drier and warmer conditions, making them dominant in postharvest scenarios. *Penicillium* spp. grows optimally at temperatures of 25–30 °C and a*_w_* levels as low as 0.78–0.80, while *Aspergillus* spp. can tolerate temperatures of 30–40 °C and a*_w_* levels as low as 0.73. These traits make drying and storage practices critical in preventing contamination [6,7].

Notably, the conditions that promote fungal growth do not always align with those that optimize mycotoxin production. For instance, *Aspergillus flavus*, a major producer of aflatoxins, grows best at 30–33 °C, but produces aflatoxins most efficiently at temperatures between 24 °C and 28 °C [8,9,10]. A study on hazelnuts showed that aflatoxin production peaked at 30 °C immediately after drying, while maximum fungal growth and aflatoxin production coincided at 35 °C after 14 days of storage [11]. Similarly, moisture levels play a crucial role: on peanuts, *A. flavus* grows optimally at a*_w_* 0.94 and 34 °C, while aflatoxin B1 production peaks at a*_w_* 0.99 and 32 °C. Matrix composition also influences contamination risk, as nutrient-rich substrates, especially those high in carbohydrates and lipids, enhance fungal growth and mycotoxin production [12,13].

Fungal contamination can occur at any stage of the supply chain. Pre-harvest contamination is often facilitated by environmental stresses such as drought, insect damage, or adverse weather conditions like rain and hail, which compromise the fruit integrity and allow fungal spores to penetrate [14]. Insects can also act as vectors for spores, further increasing contamination risks [15,16,17]. Postharvest processes, including washing, shelling, sorting, and drying, present additional contamination risks if not conducted under strict hygiene and control measures. Inefficient drying or poor sanitation of equipment can exacerbate fungal proliferation. Even during storage, suboptimal conditions, such as inadequate temperature, humidity, or aeration, can encourage fungal growth and mycotoxin production [18,19].

The presence of mycotoxins in nuts is a significant food safety concern. Commission Regulation (EU) 2023/915 establishes maximum levels for certain contaminants in food [20]. In detail, with regard to the mycotoxins analyzed in this work, the Regulation sets maximum limits for ochratoxin A (OTA) and aflatoxins in specific types of nuts. For OTA, a maximum level of 5.0 µg/kg is established for pistachios. Concerning aflatoxins, the limit values vary depending on the nut type. Specifically, for peanuts used solely as an ingredient or processed from nuts, placed on the market for the final consumer or used as an ingredient in food, the aflatoxin B_1_ (AFB1) values must not exceed 2.0 µg/kg, while the sum of AFB1, aflatoxin B_2_ (AFB2), aflatoxin G_1_ (AFG1), and aflatoxin G_2_ (AFG2) must not exceed 4.0 µg/kg. For almonds, the limit values are 8.0 and 10.0 µg/kg for AFB1 and for the sum of AFB1, AFB2, AFG1, and AFG2. For hazelnuts and Brazil nuts, the limit values are 5.0 and 10.0 µg/kg for AFB1 and for the sum of AFB1, AFB2, AFG1, and AFG2.

These metabolites, although often confined to the surface or outer layers of food products, pose serious health risks even at low concentrations due to their nephrotoxic, hepatotoxic, and neurotoxic effects. Acute mycotoxin poisoning is rare, typically occurring after short-term exposure to high doses, but chronic exposure to low concentrations over time is more common. The latter can lead to long-term health effects that remain poorly understood, often compounding other health conditions [21,22,23,24]. The heterogeneity of mycotoxin distribution within contaminated batches further complicates detection and mitigation efforts, necessitating rigorous sampling and analysis protocols, as outlined in Commission Implementing Regulation (EU) 2023/2782 [25]. In this context, guidelines have been drawn up by the European Commission [26], which should be taken into account during method validation and sample analysis, complementing the “Specific requirements for confirmatory methods” in Annex II of the aforementioned Commission Implementing Regulation (EU) 2023/2782 [25].

Preventing fungal contamination and mycotoxin production requires a comprehensive approach encompassing field management, postharvest processing, and storage practices. Pre-harvest strategies include minimizing environmental stresses and managing insect vectors, while postharvest measures focus on efficient drying, thorough sanitation, and controlled storage conditions. Regular monitoring and sampling are essential to ensure product safety and compliance with food safety standards [27,28,29,30,31].

Mycotoxin contamination represents a challenge for the Italian economy, as Italy is one of the world’s leading producers of nuts, ranking second in hazelnut production after Turkey, and is also a major producer of almonds and chestnuts. For the year 2023, nut production in Italy was 269,960 t/year, with hazelnuts and almonds as the main contributors [32]. In addition, in 2022, Italy imported $273.2 million worth of nuts from the United States [33]. In 2023, the European Union imported $6.5 billion worth of tree nuts, remaining a net importer due to demand far exceeding domestic production [34]. Italy continues to be an important exporter to the European market, particularly of shelled hazelnuts, with main destinations such as Germany, France and the United Kingdom. Thus, understanding mycotoxin occurrence in Italian nuts is relevant both for domestic consumption and for assessing risks in international trade.

The challenge of fungal contamination and mycotoxin production underscores the need for integrated preventive measures throughout the tree nut supply chain. Addressing these risks effectively is critical to safeguarding public health, ensuring product quality, and maintaining Italy’s position in the global tree nut market. Aflatoxin and ochratoxin contamination are heavily regulated by EU legislation, but the literature shows that other mycotoxins produced by species of Penicillium could occur in commercial nut samples, representing a high risk for consumers’ health.

The aim of the present study was to monitor mycotoxins in packed samples of nuts commercialized in Italy, focusing on 19 secondary metabolites from *Penicillium* spp. Samples from different matrices (peanut, almond, hazelnut and walnut), characteristics and origin were analyzed using two validated LC-MS/MS methods.

## 2. Results and Discussion

**Figure 1:**
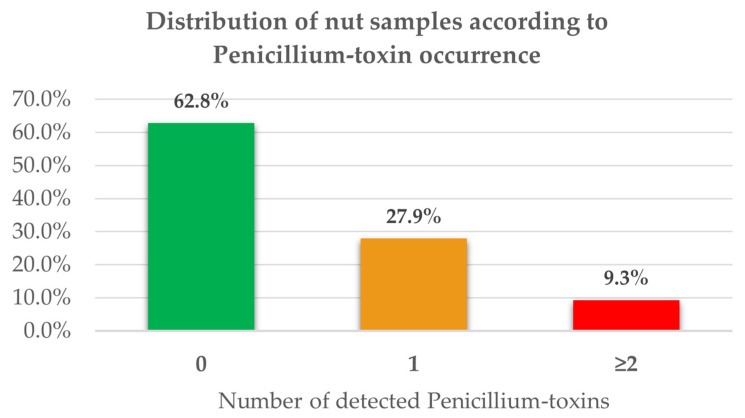
Bar chart showing the number and percentage of analyzed nut samples containing no Penicillium-toxins, one Penicillium secondary metabolite, or two or more Penicillium-toxins.

A total of 43 commercial samples, including peanuts, almonds, hazelnuts, and walnuts, were analyzed. Regarding Penicillium-toxins, 37.2% of the samples showed the presence of at least one secondary metabolite typical of this filamentous fungus, while 9.3% showed the simultaneous presence of two or more Penicillium-toxins (Figure 1 and Tables S1–S5).

The most common secondary metabolite detected among the samples was patulin, found in 14% of the nut samples analyzed (Table 1).

Patulin is produced by *Penicillium expansum*, a species of *Penicillium* commonly found on nuts. Prencipe et al. (2018) demonstrated that all seven fungal strains of *P. expansum* isolated in their research could produce patulin, only four were able to produce chaetoglobosin A, and just one produced roquefortine C [19]. Patulin thermal stability contributes to its persistence in products like nuts, which undergo heat treatments such as drying and, in some cases, roasting. This stability makes it more likely to remain present even after such processes.

**Figure 2:**
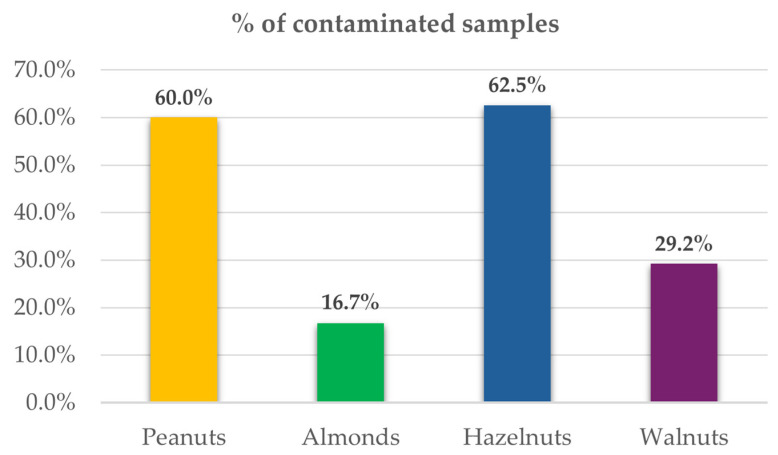
Percentage of nut samples contaminated with Penicillium-toxins.

Among the nuts investigated, hazelnuts showed the greatest prevalence of Penicillium-toxin contamination, with detection of at least one metabolite in 62.5% of the samples, followed by a slightly lower proportion of peanuts at 60.0% (Table S6, Figure 2).

Likewise, in a study conducted by Varga et al. (2013) that investigated the presence of various mycotoxins, including aflatoxins, fumonisins, enniatins, and Penicillium-toxins, in almonds, hazelnuts, pistachios, and peanuts, hazelnuts were found to have the highest incidence of mycotoxin contamination, followed by peanuts [35].

However, the sample type with the greatest variety of secondary metabolites and the highest average concentrations of these metabolites was walnuts, in which seven different metabolites were detected.

In two hazelnut samples, the presence of cyclopenol was detected, while in one walnut sample only cyclopenin was found. In two other walnut samples, both cyclopenin and cyclopenol were detected. Cyclopenin serves as a precursor to cyclopenol; in fact, when both were present, cyclopenin levels were consistently lower than cyclopenol concentrations. This same relationship was observed in the study by Spadaro et al. (2020), which examined the presence of Penicillium-toxins in hazelnuts, almonds, chestnuts, and walnuts [14]. In their research, CPL and CPN were the most frequently detected secondary metabolites, found across all matrices. Similarly to Spadaro et al. (2020), cyclopenin and cyclopenol were among the most prevalent metabolites in our study, detected in 9.3% and 7% of samples, respectively [14].

Cyclopiazonic acid was not detected in any of the samples analyzed, contrasting with the findings of the Brazilian study by Zorzete et al. (2013), where cyclopiazonic acid was detected in 60.0% and 74.3% of peanut samples from two different cultivars, with concentration ranges between approximately 288.0 µg/kg and 4918.1 µg/kg [36]. Another study, by Oyedele et al. (2017) conducted in Nigeria, found cyclopiazonic acid in 6.0% of peanut samples, with concentrations ranging from a minimum of 9.6 µg/kg to a maximum of 114 µg/kg and OTA in only 2.4% of the samples, with low concentrations, averaging about 2.0 µg/kg [37]. In contrast, research by Furlani and Soares (1996) and by Abdel-Gawad and Zohri (1993) did not detect ochratoxin A in any nut samples [38,39].

Furthermore, meleagrin, penitrem A, penicillin G and V, griseofulvin, asterric acid, citreoviridin, and sulochrin were not detected in any of the samples, aligning with the findings of Spadaro et al. (2020), who detected penitrem A and sulochrin only in chestnut samples, with no detectable concentrations of the other metabolites in chestnuts, almonds, hazelnuts, or walnuts [14].

Chaetoglobosin A was also not detected in any of the samples analyzed. However, Spadaro et al. (2020) reported concentrations of 7.6 µg/kg and 29.2 µg/kg of chaetoglobosin A in two hazelnut samples, with significantly higher concentrations found only in chestnut samples, where 62% were contaminated, reaching a maximum concentration of approximately 3763.5 µg/kg [14].

Mycophenolic acid was detected in one walnut sample at a concentration of 2.60 ± 0.50 µg/kg and in one hazelnut sample at a concentration of 2.67 ± 0.10 µg/kg. Similarly, a study by Varga et al. (2013) found mycophenolic acid in nuts, particularly in hazelnuts, but at higher levels and with greater prevalence [35]. They detected mycophenolic acid in 43.0% of the nut samples analyzed, including almonds, hazelnuts, peanuts, and pistachios. Specifically, in 18 out of 22 hazelnut samples (82.0%), they found an average contamination of approximately 700 µg/kg, reaching a maximum of around 1600 µg/kg. In 6 out of 15 peanut samples (40.0%), they observed an average concentration of 21 µg/kg, with a maximum of 60 µg/kg. Additionally, one pistachio sample out of eight contained a mycophenolic acid concentration of 13 µg/kg.

Citrinin was detected in only one sample of Italian walnuts, with a concentration of 3.07 ± 0.25 µg/kg. Similarly, studies by Abdel-Gawad and Zohri (1993) on chestnuts, almonds, cashews, hazelnuts, pistachios, and walnuts, as well as by Abdel-Hafez and Saber (1993) on hazelnuts and walnuts, found no significant citrinin levels [38,40]. Additionally, Spadaro et al. (2020) did not detect measurable amounts of citrinin in their nut samples [14].

Patulin was detected in three samples of Italian hazelnuts, two of which were roasted, and in three samples of roasted peanuts. Since patulin is a mycotoxin regulated in other food matrices under Regulation (EU) 2023/915 [20], its presence in nuts, although not currently regulated, may pose a potential risk to consumers. Patulin concentrations were higher in hazelnut samples than in peanut samples, with average concentrations of 21.88 ± 2.32 µg/kg and 3.72 ± 0.41 µg/kg, respectively. The highest concentration, 39.85 ± 2.29 µg/kg, was found in a sample of roasted and peeled Italian hazelnuts. Patulin is thermally stable, resistant to pasteurization temperatures (90 °C), and remains stable in acidic environments with a pH of around 3.5–5.5, even at 125 °C [41,42]. However, reductions in patulin levels were observed in cider after HTST (high-temperature short-time) pasteurization at 90 °C or with 90 °C treatment for 10 min [43]. In nuts, the pH is not acidic enough to preserve this secondary metabolite against heat, suggesting that roasting likely reduced but did not eliminate contamination. No patulin was detected in walnut and almond samples, consistent with studies by Abdel-Hafez and Saber (1993) and Abdel-Gawad and Zohri (1993) [38,39].

Frisvad and Samson (2004) observed the production of roquefortine C in nuts by *P. crustosum* [44], while Prencipe et al. (2018) and Garello et al. (2024) found this metabolite in chestnuts inoculated with various *Penicillium* species, including *P. expansum* and *P. crustosum* [2,19]. In Prencipe et al. (2018), roquefortine C was found in two samples of Italian walnuts and one sample of foreign almonds [19]. The highest concentration, 118.9 ± 33.50 µg/kg, was significantly higher than those detected in the other two positive samples, 1.36 ± 0.14 µg/kg and 0.99 ± 0.26 µg/kg for walnut and almond samples, respectively. This elevated concentration was found in an Italian walnut sample sold in bulk, suggesting that improper storage may have contributed to the high contamination level.

Regarding andrastin A, Prencipe et al. (2018) reported its production by *P. glandicola* inoculated in chestnuts [19]. Spadaro et al. (2020) also detected this metabolite in chestnuts and walnuts. These findings are consistent with our results, where andrastin A was found in three walnut samples, two foreign and one Italian, the latter sold in bulk, showing the highest concentration of 46.7 ± 2.60 µg/kg [14]. Once again, the fact that the highest contamination was found in unpackaged nuts reinforces the possibility that inadequate storage may promote fungal growth and/or the production of secondary metabolites.

Prencipe et al. (2018) also found that most of the fungal strains isolated from *P. crustosum*, *P. polonicum*, *P. solitum*, and *P. discolor* species produced viridicatin when inoculated in chestnuts [19]. In this study, viridicatin was detected in 21% of walnut samples (five samples), with an average concentration of 75.5 ± 18.50 µg/kg. The highest concentration, 151.4 ± 64.30 µg/kg, was found in a sample of Italian walnuts sold in bulk, while another high concentration of 124.6 ± 10.27 µg/kg was found in a sample of packaged foreign walnuts. Spadaro et al. (2020) also detected viridicatin in walnuts and almonds, but at much lower concentrations, around 3.1 µg/kg [14].

Tables S6–S9 present the percentages of contaminated samples for each matrix, along with the average, maximum, and minimum concentrations detected for each metabolite.

Penicillium toxin contamination in peanuts was evenly distributed between packed and bulk-sold samples, with a slight tendency for higher contamination in packaged peanuts (66.7%) as compared to bulk samples (50.0%).

In contrast, for hazelnuts, both bulk samples analyzed were found to be contaminated with at least one secondary metabolite, while only half of the packed samples showed contamination. A similar trend was observed for walnuts, where contamination was more prevalent in bulk samples (50.0%) as opposed to packed walnuts (25.0%). This suggests that storage methods and conditions may significantly affect product safety.

In terms of origin, 25.0% of foreign almond samples (1 out of 4) contained mycotoxins, while none of the two Italian samples showed detectable contamination, although the contamination level in the positive sample was minimal (0.99 µg/kg). For hazelnuts, while no contamination was found in foreign samples, 74% of Italian hazelnut samples showed the presence of at least one *Penicillium* spp. produced secondary metabolite. A similar pattern was observed with walnuts, where 50.0% of domestic samples were contaminated with at least one metabolite, compared to just 19.0% of foreign samples. This discrepancy may be attributed to the fact that Italy climate favors the growth of *Penicillium* species [6], while *Aspergillus* species, which require higher temperatures, are more commonly found in warmer regions [45].

The incidence of Penicillium-toxin contamination was found to be higher in walnut samples from conventional agriculture, with 35.0% of the analyzed samples showing contamination, compared to those from organic agriculture, where no detectable contamination was observed. This was despite the absence of synthetic fungicide use in organic farming. It is likely that other equally effective preventive measures against fungal attacks were implemented.

Moreover, all the organic walnuts analyzed, both shelled and unshelled, were sold in protective packaging, suggesting that this preventive measure aimed at limiting fungal growth is quite effective. Similarly, research conducted by Palumbo et al. (2015) on dried and processed nuts from conventional and organic farming revealed a higher incidence of OTA contamination in pistachios from conventional agriculture, with 7% of the samples affected, compared to those from organic farming, where no contamination was detected [46].

No aflatoxins were detected in the analyzed samples (Table S11), although the sensitivity of the method is limited due to simultaneous multi-toxin optimization. Therefore, the absence of aflatoxins should be interpreted cautiously. The absence of aflatoxins in our samples may be due to environmental and storage conditions that hinder their formation, as well as the implementation of appropriate hygienic measures that prevent contamination. In contrast to our findings, several studies reported the presence of aflatoxins in nuts. For example, a Brazilian study conduct in 2013 detected aflatoxins in 11.0% of the peanuts analyzed [36], while research conducted by Furlani and Soares (1996), also in Brazil, found aflatoxin B1 in 2 out of 54 nut samples, with concentrations ranging from approximately 10 to 26 μg/kg [39]. Additionally, they identified aflatoxin AFG1 in one sample at a concentration of around 15 μg/kg. Another study conducted in Nigeria reported average concentrations of 117 μg/kg of aflatoxin B1 in peanuts, with some samples reaching as high as 710 μg/kg. In terms of total aflatoxins, the average concentration was 216 μg/kg, with some samples peaking at 2076 μg/kg [37]. In some countries, good hygiene practices and proper storage conditions may be less rigorously applied, resulting in significantly higher levels of contamination in nuts compared to those observed in products from more developed nations. This highlights the crucial importance of promoting the adoption of good agricultural and processing practices, along with preventive and/or corrective measures to minimize the presence of mycotoxins. In these regions, particularly among small-scale producers, traditional processing methods are often used, with limited access to advanced storage systems or decontamination technologies [47]. For instance, Ozay et al. (2008) pointed out that in Turkey, the world’s largest hazelnut producer, hazelnuts are typically sun-dried, exposing them to potential adverse weather conditions and prolonging the drying time compared to mechanical drying. This extended exposure increases the risk of fungal infestation [48,49]. European legislation, as defined by Commission Regulation (EU) 2023/915, is among the most stringent worldwide [20]. Therefore, food products produced or sold within Europe must adhere to strict criteria, with inspections and random checks carried out by relevant authorities. As an example, in Italy this role is played by various local authorities, including the local health authority (*Azienda Sanitaria Locale*, ASL), Italian police protecting health—*Carabinieri per la tutela della salute, Nuclei Antisofisticazione e Sanità* (NAS), and the Central Inspectorate for Quality Protection and Fraud Prevention of Agri-food Products of the Ministry of Agriculture, Food Sovereignty and Forests (ICQRF—MASAF), to ensure compliance with current regulations [50,51]. Preventive and control measures are implemented throughout food production processes; the fact that none of the samples analyzed in this study showed detectable aflatoxin contamination demonstrates that the production process was executed correctly and that the preventive measures were effective. However, it should be noted that the relatively high limits of detection (LOD) and quantification (LOQ) of the method used, though compliant with validation criteria for complex matrices such as nuts, may not completely exclude the presence of trace amounts of aflatoxins below these thresholds. Therefore, while the results indicate an overall low contamination risk, they should be interpreted considering the analytical sensitivity of the method.

## 3. Conclusions

This study monitored the presence of Penicillium-toxins in commercial nut samples. The presence of various metabolites was detected, sometimes at high concentrations. The secondary metabolite with the highest concentration was viridicatin, found in a walnut sample at 151.40 ± 64.30 µg/kg. Additionally, several samples revealed the simultaneous presence of two or more metabolites.

A higher incidence of mycotoxin contamination was observed in Italian hazelnuts and walnuts, whereas almonds showed a higher incidence in foreign samples. Peanuts sold in packages had a higher incidence of contamination compared to loose peanuts, whereas the opposite was true for almonds and walnuts. Lastly, walnuts from conventional agriculture showed a higher incidence of contamination compared to those from organic farming.

The preventive measures implemented against *Aspergillus* section *Flavi* and mycotoxin production have proven effective, as evidenced by the absence of aflatoxins. However, the presence of Penicillium-toxins highlights the need for further preventive measures. Although these metabolites are not currently regulated in the international legislation, they exert toxic effects on humans and animals. The particularly high concentrations detected are a cause for concern.

## 4. Materials and Methods

### 4.1. Matrices and Sampling

The matrices chosen for monitoring were peanuts, almonds, hazelnuts, and walnuts (Table S12). Various types of products were purchased, sold both loose and packaged, from different sales points (supermarkets, discount stores, etc.) in Turin (Italy) and the surrounding area at various times of the year, from October 2019 to February 2020. In total, 43 samples were purchased. A sample is defined as a product with homogeneous characteristics and batch. Specifically, 5 samples of peanuts, 6 of almonds, 8 of hazelnuts, and 24 of walnuts were acquired.

Different product types were selected, including roasted peanuts in shell, almonds in shell, peeled almonds, shelled almonds, hazelnuts in shell, toasted hazelnuts in shell, shelled hazelnuts, toasted and peeled hazelnuts, walnuts in shell, shelled walnuts.

The products were further categorized based on their origin (Italian or foreign) and cultivation method (organic or conventional).

The monitoring focused on products already available on the market and thus accessible to the final consumer. At least 500 g of each type of product with homogeneous characteristics and lot were purchased. The matrices belonging to the same sample were combined into a single bag and thoroughly mixed, as prescribed by Commission Implementing Regulation (EU) 2023/2782 [25]. From these, approximately 100 g of sample (elementary sample) was taken and weighed if the product had been shelled, or approximately 200 g if the product was still enclosed in its shell or pod. Since the legal limits (although not present for Penicillium-toxins in the nut matrix) apply only to the edible part of these matrices, as stated in Commission Regulation (EU) 2023/915 [20], the sampled product was shelled or peeled (if it still had these components). Additionally, the sample was cut into pieces and then further mixed to homogenize it as much as possible. The prepared replicates, if not analyzed immediately, and the samples, once the package was opened, were stored in a freezer at −21 °C.

### 4.2. Solvents and Reagents

The HPLC and LC-MS purity grade solvents and reagents for chromatographic analyses were purchased from Sigma Aldrich (St. Louis, MO, USA), as well as the compounds used as standard (aflatoxin B1, aflatoxin B2, aflatoxin G1, aflatoxin G2, andrastin A, asterric acid, chaetoglobosin A, citreoviridin, citrinin, cyclopenin, cyclopenol, cyclopiazonic acid, griseofulvin, meleagrin, mycophenolic acid, ochratoxin A, patulin, penicillin G, penicillin V penitrem A, roquefortine C, sulochrin, and viridicatin).

### 4.3. Samples Preparations and LC-MS/MS Analyses

Identification of Penicillium-toxins and aflatoxins was performed by LC-MS/MS. The LC-MS/MS system used was an Agilent Technologies 1260 series, which included a binary pump and a vacuum degasser. It was connected to a Varian autosampler, Model 410 Prostar (Hansen Way, Palo Alto, CA, USA), featuring a 20 μL loop and coupled with a Varian 310-MS TQ Mass Spectrometer (Hansen Way, Palo Alto, CA, USA).

The extraction and separation of Penicillium-toxins followed established protocols as described by Spadaro et al. (2020), with slight adjustments made for this study [14]. Four replicates of approximately 10 g each were weighed for each sample, in polypropylene tubes. To each replicate, 15 mL of methanol acidified with 0.1% formic acid (*v*/*v*) was added for the extraction of Penicillium-toxins. The sample replicates were then subjected to high agitation using a vortex mixer for 1 min. They were then agitated using a horizontal shaker (Vibramax 100, Heidolph, Schwabach, Germany) for 30 min at 700 rpm and subsequently centrifuged (Himac CT6E centrifuge, VWR International, Radnor, PA, USA) for 10 min at 5700 rpm. Unlike the original extraction method [14], a filtration step using cellulose filters (WVR brand, pore diameter 5–13 µm, filter diameter 90 mm) was added.

A second extraction phase was carried out on the matrix with 15 mL of acetonitrile acidified with 0.1% formic acid (*v*/*v*). The sample was again agitated with the vortex and then with the horizontal shaker for half an hour, and subsequently centrifuged for 10 min at 5700 rpm. The supernatant was then filtered through cellulose. A liquid/liquid extraction with 15 mL of hexane was performed on the filtrate resulting from the two extractions to eliminate the lipid component present. Therefore, the mixture was subjected to agitation for one minute and subsequent centrifugation for 15 min at 5700 rpm. The n-hexane phase was then separated and eliminated. The extract was filtered using 0.45 µm PTFE (polytetrafluoroethylene) filters and brought to dryness using a rotary evaporator (Rotavapor LABOROTA 4000, Heidolph). The solid phase remaining after solvent evaporation was resuspended in 0.5 mL of an acetonitrile/water 50/50 (*v*/*v*) solution, centrifuged (Eppendorf Mini Spin) at 13,000 rpm, and transferred into a 1.5 mL amber vial. If the analysis was not performed immediately after the extraction process, the sample was stored in a freezer at −21 °C.

For the LC-MS/MS analyses, the chromatographic column used was a Gemini NX-C18 (150 mm × 3.0 mm L. × I.D., pore size 110 Å, particle size 3.0 µm—Phenomenex, Torrance, CA, USA). The injection volume was set to 10 µL and the flow rate to 0.3 mL/min. Solvent A was water acidified with 0.1% (*v*/*v*) of formic acid, while solvent B was acetonitrile acidified with 0.1% (*v*/*v*) of formic acid. The elution gradient was set as follows: 30% solvent B for 5 min, reaching 50% over the next 10 min and remaining constant at 50% for another 10 min, then reaching 100% in 20 min. During the subsequent 10 min of post-run, the column was equilibrated to the initial gradient percentages. Samples were ionized using an electrospray ionization (ESI) source operating in both positive and negative ion modes in different segments. For the multiple reaction monitoring (MRM) experiments, two transitions were selected for each compound (Table 2) and the collision gas (argon, Ar) pressure was set at 2.0 mbar for all the experiments.

The analysis of the four aflatoxins was carried out according to established protocols outlined by Valente et al. (2020), with minor modifications for this study [11]. Due to the photosensitivity of aflatoxins, care was taken to avoid exposure of the samples to sunlight as much as possible, in accordance with Commission Regulation (EU) 2023/915 [20]. Four replicates of approximately 12 g each were weighed, and 30 mL of an acetone-water solution (60/40, *v*/*v*) was added to each. The samples were then subjected to high agitation for one minute, followed by sonication for 30 min using an Ultrasonic Cleaner (VWR).

After 15 min of centrifugation, the sample extracts were filtered using Whatman No. 4 paper filters with a porosity of 8–12 µm. They were further filtered through a syringe filter with a 0.2 µm polypropylene membrane, after a brief centrifugation to settle any precipitated solids. From the filtrate, 10 mL was taken and mixed with 10 mL of ethyl acetate for liquid–liquid extraction, along with 1.5 g of sodium chloride (NaCl). The samples were then vortexed for one minute and centrifuged for 15 min to separate the two phases. The supernatant, containing the analytes in the ethyl acetate, was collected and transferred to a flask. The liquid–liquid extraction was repeated twice.

The combined extract was evaporated to dryness using a rotary evaporator, then reconstituted in 1 mL of 30% (*v*/*v*) acetonitrile, centrifuged, and transferred to a 1.5 mL glass vial. The vial was stored in a freezer at −21 °C until instrumental analysis.

The column used was a Kinetex C18 (100 mm × 2.1 mm L. × I.D., pore size 100 Å, particle size 2.6 µm—Phenomenex, Torrance, CA, USA). The injection volume was set to 10 μL and the flow rate to 0.2 mL/min. Solvent A was water acidified with 0.1% formic acid, while solvent B was acetonitrile acidified with 0.1% formic acid. The analysis was conducted in isocratic condition at 30% B for 10 min.

The samples were ionized using an electrospray ionization (ESI) source operating in positive ion mode. For the multiple reaction monitoring (MRM) experiments, two transitions were selected for each compound (Table S13), and the collision gas (Ar) pressure was set to 2.0 mbar for all experiments.

### 4.4. Validation of Analytical Parameters of Penicillium-Toxins

The method was validated in compliance with EN ISO/IEC 17025:2017 [52] and the performance criteria outlined in Commission Implementing Regulation (EU) 2023/2782 [24,25]. Validation was conducted for 19 toxic metabolites produced by *Penicillium* spp., assessing key parameters such as linearity range, limits of quantification (LOQ) and detection (LOD), recovery, matrix effect (ME), specificity, and selectivity, as previously described in a previous paper [14]. To confirm method robustness, additional validation tests were performed in this study. Blank nut matrices were fortified at three concentration levels (20, 50, and 100 ng/g) and analyzed in triplicate for each level. According to Commission Regulation (EC) No 401/2006, typically a recovery within the range 70–110% is required [53]. The mean recoveries for compounds were from 64.6% (RoqC) to 104.2% (GRI) for hazelnuts, 68.2% (AndA) to 98.1% (CPA) for walnuts and 66.3% (PenA) to 96.2% (CVD) for almonds. Intra-day and inter-day precision were assessed through replicate injections on the same and consecutive days, yielding satisfactory repeatability and reproducibility values (coefficient variability values, CV ≤ 15%).

### 4.5. Validation of Analytical Parameters of Aflatoxins

The linearity (R^2^) for each analyte was evaluated by injecting pure standard solutions in the matrix at various concentration levels. The limit of detection (LOD) and the limit of quantification (LOQ) were also calculated, and the recovery of the analysis was assessed by injecting solutions of the various standards into the matrix (Table S14). Repeatability and reproducibility of the method were also evaluated by performing multiple injections of the same standard solution under the same conditions and on different days [14]. The method showed good accuracy, with recoveries between 75% and 109% and repeatability and reproducibility within acceptable limits (CV ≤ 15%), consistent with EU performance criteria.

Although the LOQs of the method for aflatoxins are above the maximum levels set by Commission Regulation (EU) 2023/915 [20], the method was primarily optimized for simultaneous detection of multiple mycotoxins, including Penicillium-toxins. Therefore, the data on aflatoxins are reported for completeness but are not intended for regulatory compliance evaluation.

### 4.6. Statistical Treatment of Non-Detects

Non-detects are left-censored data, meaning their true value lies below the LOD or LOQ but is unknown. In this study, values < LOD were replaced with half the LOD, and values < LOQ with half the LOQ. This simple approach retains all samples while minimizing bias. All statistical analyses were performed on the adjusted dataset.

## Figures and Tables

**Table 1:** Percentage of contaminated samples, average, minimum, and maximum concentration of Penicillium-toxins in nuts (Tables S6–S10).

Mycotoxin	N of Samples Analyzed	N of Positive Samples	% of Contaminated Samples	Average ^a^ (μg/kg) ± SD	Minimum ^b^ (μg/kg) ± SD	Maximum ^c^ (μg/kg) ± SD
AndA	43	3	7.0%	21.20 ± 22.57	3.79 ± 0.76	46.70 ± 2.60
ChA	43	0	0	-	-	-
CIT	43	1	2.3%	3.07 ± 0.25	3.07 ± 0.25	3.07 ± 0.25
CPN	43	3	7.0%	3.57 ± 2.13	1.47 ± 0.04	5.72 ± 0.80
CPL	43	4	9.3%	31.04 ± 35.84	4.70 ± 0.66	83.80 ± 3.80
CPA	43	0	0	-	-	-
MEL	43	0	0	-	-	-
MPA	43	2	4.7%	2.64 ± 0.05	2.60 ± 0.50	2.67 ± 0.10
OTA	43	0	0	-	-	-
PAT	43	6	14.0%	12.80 ± 14.34	1.18 ± 0.18	39.85 ± 2.29
PenA	43	0	0	-	-	-
RoqC	43	3	7.0%	40.42 ± 67.97	0.99 ± 0.26	118.90 ± 33.50
VIR	43	5	11.6%	75.51 ± 60.33	8.61 ± 1.18	151.40 ± 64.30
PenG	43	0	0	-	-	-
PenV	43	0	0	-	-	-
GRI	43	0	0	-	-	-
AsA	43	0	0	-	-	-
CVD	43	0	0	-	-	-
SUL	43	0	0	-	-	-

^a^ Average of the concentrations detected for that metabolite; ^b^ Highest concentration detected; ^c^ Lowest concentration detected. SD: standard deviation. AndA: andrastina A, ChA: Chaetoglobosin A, CIT: citrinin, CPN: cyclopenin, CPL: cyclopenol, CPA: cyclopiazonic acid, MEL: meleagrin, MPA: mycophenolic acid, OTA: ochratoxin A, PAT: patulin, PenA: penitram A, RoqC: roquefortina C, VIR: viridicatin, PenG: penicillin G, PenV: penicillin V, GRI: griseofulvin, AsA: asterric acid, CVD: citreoviridine, SUL: sulochrin.

**Table 2:** Precursor and product ions with collision energy (eV) for the mycotoxins evaluated by mass spectrometric analyses. The product ions chosen for quantification are shown in bold.

Compound	Abbreviation	Formula	Retention Time (Minutes)	Precursor Ion	Product Ions (Collision Energy, eV)
Andrastin A	AndA	C_28_H_38_O_7_	34.61	487 (+)	243(22)/**427(12)**
Asterric acid	AsA	C_17_H_16_O_8_	22.02	347 (−)	**149(12)**/256(16)
Chaetoglobosin A	ChA	C_32_H_36_N_2_O_5_	29.25	529 (+)	**130(28)**/511(12)
Citreoviridin	CVD	C_23_H_30_O_6_	21.94	403 (+)	**139(24)**/297(16)
Citrinin	CIT	C_13_H_14_O_5_	20.81	251 (+)	233(16)/**205(20)**
Cyclopenin	CPN	C_17_H_14_N_2_O_3_	14.09	295 (+)	**146(24)**/177(12)
Cyclopenol	CPL	C_17_H_14_N_2_O_4_	6.68	311 (+)	**146(26)**/177(14)
Cyclopiazonic acid	CPA	C_20_H_20_N_2_O_3_	37.06	337 (+)	182(18)/**196(24)**
Griseofulvin	GRI	C_17_H_17_C_11_O_6_	21.32	353 (+)	165(22)/**215(20)**
Meleagrin	MEL	C_23_H_23_N_5_O_4_	6.46	434 (+)	**403(14)**/334(22)
Mycophenolic acid	MPA	C_17_H_20_O_6_	21.2	321 (+)	159(38)/**207(22)**
Ochratoxin A	OTA	C_20_H_18_ClNO_6_	28.63	404 (+)	**239(26)**/221(36)
Patulin	PAT	C_7_H_6_O_4_	2.54	153 (−)	**109(8)**/81(12)
Penicillin G	PenG	C_16_H_18_N_2_O_4_S	12.01	335 (+)	202(24)/**217(16)**
Penicillin V	PenV	C_16_H_18_N_2_O_5_S	15.29	351 (+)	**229(16)**/257(17)
Penitrem A	PenA	C_37_H_44_ClNO_6_	42.95	634 (+)	**558(18)**/616(12)
Roquefortine C	RoqC	C_22_H_23_N_5_O_2_	3.09	390 (+)	**193(28)**/322(20)
Sulochrin	SUL	C_17_H_16_O_7_	14.02	331 (−)	**149(16)**/299(14)
Viridicatin	VIR	C_15_H_11_NO_2_	21.48	238 (+)	220(16)/**165(30)**

## Data Availability

The original contributions presented in this study are included in the article/Supplementary Materials. Further inquiries can be directed to the corresponding author(s).

## Supplementary Materials

The following supporting information can be downloaded at: https://www.mdpi.com/article/10.3390/toxins18010012/s1, Table S1: Presence of Penicillium-toxins in peanuts; Table S2: Presence of Penicillium-toxins in almonds; Table S3: Presence of Penicillium-toxins in hazelnuts; Table S4: Presence of Penicillium-toxins in walnuts; Table S5: Distribution of nut samples according to the number of detected Penicillium-toxins; Table S6: Percentage of nut samples contaminated with Penicillium-toxins; Table S7: Percentage of peanut samples contaminated by Penicillium-toxins, along with the average, minimum, maximum, and median concentrations; Table S8: Percentage of almond samples contaminated by Penicillium-toxins, along with the average, minimum, maximum, and median concentrations; Table S9: Percentage of hazelnut samples contaminated by Penicillium-toxins, along with the average, minimum, maximum, and median concentrations; Table S10: Percentage of walnut samples contaminated by Penicillium-toxins, along with the average, minimum, maximum, and median concentrations; Table S11: Presence of aflatoxins in the analyzed samples of peanuts, almonds, hazelnuts, and walnuts; Table S12: List of samples divided according to the matrix; Table S13. Precursor and product ions with collision energy (eV) for the aflatoxins evaluated by mass spectrometric analyses. The product ions chosen for quantification are shown in bold; Table S14. Recovery, LOD, LOQ, and R^2^ for each matrix.
