# Supplementary material for: Detection of Penicillium-Toxins in Nuts Commercialized in Italy Through LC-MS/MS Analyses
Source: Toxins (Basel). 2025 Dec 24;18(1):12. doi: 10.3390/toxins18010012 (PMC12845813; doi:10.3390/toxins18010012)
Supplement: Supplementary file 1 [file toxins-18-00012-s001.zip › toxins-3954502-supplementary.pdf]

Supplementary Materials: Detection of Penicillium-Toxins in Nuts Commercialized in Italy through LC-MS/MS Analyses

Fabio Buonsenso, Giovanna Roberta Meloni and Davide Spadaro

Content.

**Table S1.** Presence of Penicillium-toxins in peanuts.	**2**
**Table S2.** Presence of Penicillium-toxins in almonds.	**2**
**Table S3.** Presence of Penicillium-toxins in hazelnuts.	**3**
**Table S4.** Presence of Penicillium-toxins in walnuts.	**4,5**
**Table S5.** Distribution of nut samples according to the number of detected Penicillium-toxins.	**6**
**Table S6.** Percentage of nut samples contaminated with Penicillium-toxins.	**6**
**Table S7**. Percentage of peanut samples contaminated by Penicillium-toxins, along with the average, minimum, maximum, and median concentrations.	**7**
**Table S8.** Percentage of almond samples contaminated by Penicillium-toxins, along with the average, minimum, maximum, and median concentrations.	**8**
**Table S9.** Percentage of hazelnut samples contaminated by Penicillium-toxins, along with the average, minimum, maximum, and median concentrations.	**9**
**Table S10.** Percentage of walnut samples contaminated by Penicillium-toxins, along with the average, minimum, maximum, and median concentrations.	**10**
**Table S11.** Presence of aflatoxins in the analyzed samples of peanuts, almonds, hazelnuts, and walnuts.	**11,12**
**Table S12.** List of samples divided according to the matrix.	**13,14**
**Table S13.** Precursor and product ions with collision energy (eV) for the aflatoxins evaluated by mass spectrometric analyses. The product ions chosen for quantification are shown in bold.	**14**
**Table S14.** Recovery, LOD, LOQ, and R^2 for each matrix.	**15**

https://doi.org/10.3390/toxins18010012

Table S1. Presence of Penicillium-toxins in peanuts. SD: standard deviation.

	[AndA] µg/kg± SD[b]	[ChA] µg/kg ± SD	[CIT] µg/kg ± SD	[CPN] µg/kg ± SD	[CPL] µg/kg ± SD	[CPA] µg/kg ± SD	[MEL] µg/kg ± SD	[MPA] µg/kg± SD	[OTA] µg/kg ± SD	[PAT] µg/kg ± SD	[PenA] µg/kg ± SD	[RoqC] µg/kg ± SD	[VIR] µg/kg ± SD	[PenG] µg/kg ± SD	[PenV] µg/kg ± SD	[GRI] µg/kg ± SD	[AsA] µg/kg ± SD	[CIV] µg/kg ± SD	[SUL] µg/kg ± SD
**ATCE**	<LOD	<LOD	<LOD	<LOD	<LOD	<LOD	<LOD	<LOD	<LOD	3.00 ± 0.29	<LOD	<LOD	<LOD	<LOD	<LOD	<LOD	<LOD	<LOD	<LOD
**ATCE1**	<LOD	<LOD	<LOD	<LOD	<LOD	<LOD	<LOD	<LOD	<LOD	6.98 ± 0.77	<LOD	<LOD	<LOD	<LOD	<LOD	<LOD	<LOD	<LOD	<LOD
**ATCE2**	<LOD	<LOD	<LOD	<LOD	<LOD	<LOD	<LOD	<LOD	<LOD	<LOD	<LOD	<LOD	<LOD	<LOD	<LOD	<LOD	<LOD	<LOD	<LOD
**ATCE3**	<LOD	<LOD	<LOD	<LOD	<LOD	<LOD	<LOD	<LOD	<LOD	1.18 ± 0.18	<LOD	<LOD	<LOD	<LOD	<LOD	<LOD	<LOD	<LOD	<LOD
**ATCE4**	<LOD	<LOD	<LOD	<LOD	<LOD	<LOD	<LOD	<LOD	<LOD	<LOD	<LOD	<LOD	<LOD	<LOD	<LOD	<LOD	<LOD	<LOD	<LOD

Table S2. Presence of Penicillium-toxins in almonds. SD: standard deviation

	[AndA] µg/kg± SD[b]	[ChA] µg/kg ± SD	[CIT] µg/kg ± SD	[CPN] µg/kg ± SD	[CPL] µg/kg ± SD	[CPA] µg/kg ± SD	[MEL] µg/kg ± SD	[MPA] µg/kg± SD	[OTA] µg/kg ± SD	[PAT] µg/kg ± SD	[PenA] µg/kg ± SD	[RoqC] µg/kg ± SD	[VIR] µg/kg ± SD	[PenG] µg/kg ± SD	[PenV] µg/kg ± SD	[GRI] µg/kg ± SD	[AsA] µg/kg ± SD	[CIV] µg/kg ± SD	[SUL] µg/kg ± SD
**MGCI1**	<LOD	<LOD	<LOD	<LOD	<LOD	<LOD	<LOD	<LOD	<LOD	<LOD	<LOD	<LOD	<LOD	<LOD	<LOD	<LOD	<LOD	<LOD	<LOD
**MPCE**	<LOD	<LOD	<LOD	<LOD	<LOD	<LOD	<LOD	<LOD	<LOD	<LOD	<LOD	<LOD	<LOD	<LOD	<LOD	<LOD	<LOD	<LOD	<LOD
**MS2CI**	<LOD	<LOD	<LOD	<LOD	<LOD	<LOD	<LOD	<LOD	<LOD	<LOD	<LOD	<LOD	<LOD	<LOD	<LOD	<LOD	<LOD	<LOD	<LOD
**MSCE1**	<LOD	<LOD	<LOD	<LOD	<LOD	<LOD	<LOD	<LOD	<LOD	<LOD	<LOD	<LOD	<LOD	<LOD	<LOD	<LOD	<LOD	<LOD	<LOD
**MSCE2**	<LOD	<LOD	<LOD	<LOD	<LOD	<LOD	<LOD	<LOD	<LOD	<LOD	<LOD	<LOD	<LOD	<LOD	<LOD	<LOD	<LOD	<LOD	<LOD
**MSCE3**	<LOD	<LOD	<LOD	<LOD	<LOD	<LOD	<LOD	<LOD	<LOD	<LOD	<LOD	0.99 ± 0.26	<LOD	<LOD	<LOD	<LOD	<LOD	<LOD	<LOD

https://doi.org/10.3390/toxins18010012

Table S3. Presence of Penicillium-toxins in hazelnuts. SD: standard deviation

	[AndA] µg/kg± SD[b]	[ChA] µg/kg ± SD	[CIT] µg/kg ± SD	[CPN] µg/kg ± SD	[CPL] µg/kg ± SD	[CPA] µg/kg ± SD	[MEL] µg/kg ± SD	[MPA] µg/kg± SD	[OTA] µg/kg ± SD	[PAT] µg/kg ± SD	[PenA] µg/kg ± SD	[RoqC] µg/kg ± SD	[VIR] µg/kg ± SD	[PenG] µg/kg ± SD	[PenV] µg/kg ± SD	[GRI] µg/kg ± SD	[AsA] µg/kg ± SD	[CVD] µg/kg ± SD	[SUL] µg/kg ± SD
**NGCI**	<LOD	<LOD	<LOD	<LOD	<LOD	<LOD	<LOD	<LOD	<LOD	8.94 ± 2.04	<LOD	<LOD	<LOD	<LOD	<LOD	<LOD	<LOD	<LOD	<LOD
**NGTCE**	<LOD	<LOD	<LOD	<LOD	<LOD	<LOD	<LOD	<LOD	<LOD	<LOD	<LOD	<LOD	<LOD	<LOD	<LOD	<LOD	<LOD	<LOD	<LOD
**NGTCI**	<LOD	<LOD	<LOD	<LOD	<LOD	<LOD	<LOD	<LOD	<LOD	<LOD	<LOD	<LOD	<LOD	<LOD	<LOD	<LOD	<LOD	<LOD	<LOD
**NGTCI1**	<LOD	<LOD	<LOD	<LOD	<LOD	<LOD	<LOD	<LOD	<LOD	16.85 ± 2.65	<LOD	<LOD	<LOD	<LOD	<LOD	<LOD	<LOD	<LOD	<LOD
**NSCI**	<LOD	<LOD	<LOD	<LOD	<LOD	<LOD	<LOD	<LOD	<LOD	<LOD	<LOD	<LOD	<LOD	<LOD	<LOD	<LOD	<LOD	<LOD	<LOD
**NTCI**	<LOD	<LOD	<LOD	<LOD	<LOD	<LOD	<LOD	<LOD	<LOD	39.85 ± 2.29	<LOD	<LOD	<LOD	<LOD	<LOD	<LOD	<LOD	<LOD	<LOD
**NOCC1**	<LOD	<LOD	<LOD	<LOD	21.45 ± 6.40	<LOD	<LOD	<LOD	<LOD	<LOD	<LOD	<LOD	<LOD	<LOD	<LOD	<LOD	<LOD	<LOD	<LOD
**NOCC2**	<LOD	<LOD	<LOD	<LOD	14.20 ± 2.90	<LOD	<LOD	2.67 ± 0.10	<LOD	<LOD	<LOD	<LOD	<LOD	<LOD	<LOD	<LOD	<LOD	<LOD	<LOD

https://doi.org/10.3390/toxins18010012

Table S4. Presence of Penicillium-toxins in walnuts. SD: standard deviation

	[AndA] µg/kg± SD[b]	[ChA] µg/kg ± SD	[CIT] µg/kg ± SD	[CPN] µg/kg ± SD	[CPL] µg/kg ± SD	[CPA] µg/kg ± SD	[MEL] µg/kg ± SD	[MPA] µg/kg± SD	[OTA] µg/kg ± SD	[PAT] µg/kg ± SD	[PenA] µg/kg ± SD	[RoqC] µg/kg ± SD	[VIR] µg/kg ± SD	[PenG] µg/kg ± SD	[PenV] µg/kg ± SD	[GRI] µg/kg ± SD	[AsA] µg/kg ± SD	[CVD] µg/kg ± SD	[SUL] µg/kg ± SD
**OGBE**	<LOD	<LOD	<LOD	<LOD	<LOD	<LOD	<LOD	<LOD	<LOD	<LOD	<LOD	<LOD	<LOD	<LOD	<LOD	<LOD	<LOD	<LOD	<LOD
**OGBE1**	<LOD	<LOD	<LOD	<LOD	<LOD	<LOD	<LOD	<LOD	<LOD	<LOD	<LOD	<LOD	<LOD	<LOD	<LOD	<LOD	<LOD	<LOD	<LOD
**OGCE**	<LOD	<LOD	<LOD	<LOD	<LOD	<LOD	<LOD	<LOD	<LOD	<LOD	<LOD	<LOD	<LOD	<LOD	<LOD	<LOD	<LOD	<LOD	<LOD
**OGCE1**	<LOD	<LOD	<LOD	<LOD	<LOD	<LOD	<LOD	<LOD	<LOD	<LOD	<LOD	<LOD	8.61 ± 1.18	<LOD	<LOD	<LOD	<LOD	<LOD	<LOD
**OGCE2**	13.11 ± 0.73	<LOD	<LOD	3.53 ± 0.58	4.70 ± 0.66	<LOD	<LOD	<LOD	<LOD	<LOD	<LOD	<LOD	124.69 ± 10.27	<LOD	<LOD	<LOD	<LOD	<LOD	<LOD
**OGCE3**	<LOD	<LOD	<LOD	<LOD	<LOD	<LOD	<LOD	<LOD	<LOD	<LOD	<LOD	<LOD	<LOD	<LOD	<LOD	<LOD	<LOD	<LOD	<LOD
**OGCE4**	<LOD	<LOD	<LOD	<LOD	<LOD	<LOD	<LOD	<LOD	<LOD	<LOD	<LOD	<LOD	<LOD	<LOD	<LOD	<LOD	<LOD	<LOD	<LOD
**OGCE5**	<LOD	<LOD	<LOD	<LOD	<LOD	<LOD	<LOD	<LOD	<LOD	<LOD	<LOD	<LOD	<LOD	<LOD	<LOD	<LOD	<LOD	<LOD	<LOD
**OGCE6**	<LOD	<LOD	<LOD	<LOD	<LOD	<LOD	<LOD	<LOD	<LOD	<LOD	<LOD	<LOD	<LOD	<LOD	<LOD	<LOD	<LOD	<LOD	<LOD
**OGCE7**	<LOD	<LOD	<LOD	<LOD	<LOD	<LOD	<LOD	<LOD	<LOD	<LOD	<LOD	<LOD	<LOD	<LOD	<LOD	<LOD	<LOD	<LOD	<LOD
**OGCE8**	3.79 ± 0.76	<LOD	<LOD	<LOD	<LOD	<LOD	<LOD	<LOD	<LOD	<LOD	<LOD	<LOD	<LOD	<LOD	<LOD	<LOD	<LOD	<LOD	<LOD
**OGCI1**	<LOD	<LOD	<LOD	<LOD	<LOD	<LOD	<LOD	<LOD	<LOD	<LOD	<LOD	<LOD	<LOD	<LOD	<LOD	<LOD	<LOD	<LOD	<LOD
**OGCI2**	<LOD	<LOD	<LOD	<LOD	<LOD	<LOD	<LOD	<LOD	<LOD	<LOD	<LOD	<LOD	<LOD	<LOD	<LOD	<LOD	<LOD	<LOD	<LOD
**OGCI3**	<LOD	<LOD	<LOD	<LOD	<LOD	<LOD	<LOD	<LOD	<LOD	<LOD	<LOD	<LOD	<LOD	<LOD	<LOD	<LOD	<LOD	<LOD	<LOD
**OGCI4**	<LOD	<LOD	<LOD	<LOD	<LOD	<LOD	<LOD	<LOD	<LOD	<LOD	<LOD	<LOD	<LOD	<LOD	<LOD	<LOD	<LOD	<LOD	<LOD

https://doi.org/10.3390/toxins18010012

**OGCI5**	<LOD	<LOD	3.07 ± 0.25	<LOD	<LOD	<LOD	<LOD	<LOD	<LOD	<LOD	<LOD	<LOD	<LOD	<LOD	<LOD	<LOD	<LOD	<LOD
**OGCI6**	<LOD	<LOD	<LOD	<LOD	<LOD	<LOD	<LOD	<LOD	<LOD	<LOD	<LOD	<LOD	56.77 ± 12.71	<LOD	<LOD	<LOD	<LOD	<LOD	<LOD

Table S4. (continued)

	**[AndA] μg/kg± SD[b]**	**[ChA] μg/kg ± SD**	**[CIT] μg/kg ± SD**	**[CPN] μg/kg ± SD**	**[CPL] μg/kg ± SD**	**[CPA] μg/kg ± SD**	**[MEL] μg/kg ± SD**	**[MPA] μg/kg± SD**	**[OTA] μg/kg ± SD**	**[PAT] μg/kg ± SD**	**[PenA] μg/kg ± SD**	**[RoqC] μg/kg ± SD**	**[VIR] μg/kg ± SD**	**[PenG] μg/kg ± SD**	**[PenV] μg/kg ± SD**	**[GRI] μg/kg ± SD**	**[AsA] μg/kg ± SD**	**[CVD] μg/kg ± SD**	**[SUL] μg/kg ± SD**
**OGCI7**	<LOD	<LOD	<LOD	1.47 ± 0.04	<LOD	<LOD	<LOD	<LOD	<LOD	<LOD	<LOD	1.36 ± 0.14	36.09 ± 4.03	<LOD	<LOD	<LOD	<LOD	<LOD	<LOD
**OS1BE**	<LOD	<LOD	<LOD	<LOD	<LOD	<LOD	<LOD	<LOD	<LOD	<LOD	<LOD	<LOD	<LOD	<LOD	<LOD	<LOD	<LOD	<LOD	<LOD
**OSBE1**	<LOD	<LOD	<LOD	<LOD	<LOD	<LOD	<LOD	<LOD	<LOD	<LOD	<LOD	<LOD	<LOD	<LOD	<LOD	<LOD	<LOD	<LOD	<LOD
**OSCE**	<LOD	<LOD	<LOD	<LOD	<LOD	<LOD	<LOD	<LOD	<LOD	<LOD	<LOD	<LOD	<LOD	<LOD	<LOD	<LOD	<LOD	<LOD	<LOD
**OSCE1**	<LOD	<LOD	<LOD	<LOD	<LOD	<LOD	<LOD	<LOD	<LOD	<LOD	<LOD	<LOD	<LOD	<LOD	<LOD	<LOD	<LOD	<LOD	<LOD
**OSCE2**	<LOD	<LOD	<LOD	<LOD	<LOD	<LOD	<LOD	<LOD	<LOD	<LOD	<LOD	<LOD	<LOD	<LOD	<LOD	<LOD	<LOD	<LOD	<LOD
**NOC1**	46.70 ± 2.60	<LOD	<LOD	5.72 ± 0.80	83.80 ± 3.80	<LOD	<LOD	2.60± 0.50	<LOD	<LOD	<LOD	118.90 ± 33.50	151.40 ± 64.30	<LOD	<LOD	<LOD	<LOD	<LOD	<LOD

Table S5. Distribution of nut samples according to the number of detected Penicillium-toxins.

Number of detected Penicillium-toxins	Number of samples	Percentage (%)
0	27	62.8
1	12	27.9
≥2	4	9.3
**Total**	**43**	**100**

Table S6. Percentage of dried fruit samples contaminated with Penicillium-toxins.

Samples	N of samples analyzed	N of positive samples	% of contaminated samples
Peanuts	5	3	60.0
Almonds	6	1	16.7
Hazelnuts	8	5	62.5
Walnuts	24	7	29.2

https://doi.org/10.3390/toxins18010012

Table S7. Percentage of peanut samples contaminated by Penicillium-toxins, along with the average, minimum, maximum, and median concentrations. a: average concentration detected for that metabolite; b: median concentration of that metabolite; c: highest concentration detected; d: lowest concentration detected; SD: standard deviation.

**Peanut**	**N of samples analyzed**	**N of positive samples**	**% of contaminated samples**	**Average[a] (µg/kg) ± SD**	**Median[b]**	**Maximum concentration[c] (µg/kg) ± SD**	**Minimum concentration[d] (µg/kg) ± SD**
AndA	5	0	0	-	-	-	-
ChA	5	0	0	-	-	-	-
CIT	5	0	0	-	-	-	-
CPN	5	0	0	-	-	-	-
CPL	5	0	0	-	-	-	-
CPA	5	0	0	-	-	-	-
MEL	5	0	0	-	-	-	-
MPA	5	0	0	-	-	-	-
OTA	5	0	0	-	-	-	-
PAT	5	3	60.0%	3.72±0.41	3.00	6.98±0.77	1.18±0.18
PenA	5	0	0	-	-	-	-
RoqC	5	0	0	-	-	-	-
VIR	5	0	0	-	-	-	-
PenG	5	0	0	-	-	-	-
PenV	5	0	0	-	-	-	-
GRI	5	0	0	-	-	-	-
AsA	5	0	0	-	-	-	-
CVD	5	0	0	-	-	-	-
SUL	5	0	0	-	-	-	-

https://doi.org/10.3390/toxins18010012

Table S8. Percentage of almond samples contaminated by Penicillium-toxins, along with the average, minimum, maximum, and median concentrations. a: average concentration detected for that metabolite; b: median concentration of that metabolite; c: highest concentration detected; d: lowest concentration detected; SD: standard deviation.

**Almond**	**N of samples analyzed**	**N of positive samples**	**% of contaminated samples**	**Average[a] (µg/kg) ± SD**	**Median[b]**	**Maximum concentration [c] (µg/kg) ± SD**	**Minimum concentration [d] (µg/kg) ± SD**
AndA	6	0	0	-	-	-	-
ChA	6	0	0	-	-	-	-
CIT	6	0	0	-	-	-	-
CPN	6	0	0	-	-	-	-
CPL	6	0	0	-	-	-	-
CPA	6	0	0	-	-	-	-
MEL	6	0	0	-	-	-	-
MPA	6	0	0	-	-	-	-
OTA	6	0	0	-	-	-	-
PAT	6	0	0	-	-	-	-
PenA	6	0	0	-	-	-	-
RoqC	6	1	16.7%	0.99±0.26	0.99±0.26	0.99±0.26	0.99±0.26
VIR	6	0	0	-	-	-	-
PenG	6	0	0	-	-	-	-
PenV	6	0	0	-	-	-	-
GRI	6	0	0	-	-	-	-
AsA	6	0	0	-	-	-	-
CVD	6	0	0	-	-	-	-
SUL	6	0	0	-	-	-	-

https://doi.org/10.3390/toxins18010012

Table S9. Percentage of hazelnut samples contaminated by Penicillium-toxins, along with the average, minimum, maximum, and median concentrations. a: average concentration detected for that metabolite; b: median concentration of that metabolite; c: highest concentration detected; d: lowest concentration detected; SD: standard deviation.

Hazelnut	N of samples analyzed	N of positive samples	% of contaminated samples	Average[a] (µg/kg) ± SD	Median[b]	Maximum concentration[c] (µg/kg) ± SD	Minimum concentration[d] (µg/kg) ± SD
AndA	8	0	0	-	-	-	-
ChA	8	0	0	-	-	-	-
CIT	8	0	0	-	-	-	-
CPN	8	0	0	-	-	-	-
CPL	8	2	25.0%	17.82±4.65	17.83	21.45±6.40	14.20±2.90
CPA	8	0	0	-	-	-	-
MEL	8	0	0	-	-	-	-
MPA	8	1	12.5%	2.67±0.10	2.67	2.67±0.10	2.67±0.10
OTA	8	0	0	-	-	-	-
PAT	8	3	37.5%	21.88±2.33	16.85	39.85±2.29	8.94±2.04
PenA	8	0	0	-	-	-	-
RoqC	8	0	0	-	-	-	-
VIR	8	0	0	-	-	-	-
PenG	8	0	0	-	-	-	-
PenV	8	0	0	-	-	-	-
GRI	8	0	0	-	-	-	-
AsA	8	0	0	-	-	-	-
CVD	8	0	0	-	-	-	-
SUL	8	0	0	-	-	-	-

https://doi.org/10.3390/toxins18010012

Table S10. Percentage of walnut samples contaminated by Penicillium-toxins, along with the average, minimum, maximum, and median concentrations. a: average concentration detected for that metabolite; b: median concentration of that metabolite; c: highest concentration detected; d: lowest concentration detected; SD: standard deviation.

**Walnut**	**N of samples analyzed**	**N of positive samples**	**% of contaminated samples**	**Average[a] (µg/kg) ± SD**	**Median[b]**	**Maximum concentration[c] (µg/kg) ± SD**	**Minimum concentration[d] (µg/kg) ± SD**
AndA	24	3	12.5%	21.20±1.37	13.11	46.70±2.60	3.79±0.76
ChA	24	0	0	-	-	-	-
CIT	24	1	4.2%	3.07±0.25	3.07	3.07±0.25	3.07±0.25
CPN	24	3	12.5%	3.57±0.48	3.53	5.72±0.80	1.47±0.04
CPL	24	2	8.3%	44.25±2.23	44.25	83.80±3.80	4.70±0.66
CPA	24	0	0	-	-	-	-
MEL	24	0	0	-	-	-	-
MPA	24	1	4.2%	2.60±0.50	2.60	2.60±0.50	2.60±0.50
OTA	24	0	0	-	-	-	-
PAT	24	0	0	-	-	-	-
PenA	24	0	0	-	-	-	-
RoqC	24	2	8.3%	60.13±16.82	60.13	118.90±33.50	1.36±0.14
VIR	24	5	20.8%	75.51±18.50	56.77	151.40±64.30	8.61±1.18
PenG	24	0	0	-	-	-	-
PenV	24	0	0	-	-	-	-
GRI	24	0	0	-	-	-	-
AsA	24	0	0	-	-	-	-
CVD	24	0	0	-	-	-	-
SUL	24	0	0	-	-	-	-

Table S11. Presence of aflatoxins in the analyzed samples of peanuts, almonds, hazelnuts, and walnuts. SD: standard deviation; LOD: limit of detection.

**Samples**	**[AFB1] µg/kg ± SD**	**[TOTAL AFs] µg/kg ± SD**
**Peanuts**		
ATCE	<LOD	<LOD
ATCE1	<LOD	<LOD
ATCE2	<LOD	<LOD
ATCE3	<LOD	<LOD
ATCE4	<LOD	<LOD
**Almonds**		
MGCI1	<LOD	<LOD
MPCE	<LOD	<LOD
MS2CI	<LOD	<LOD
MSCE1	<LOD	<LOD
MSCE2	<LOD	<LOD
MSCE3	<LOD	<LOD
**Hazelnuts**		
NGCI	<LOD	<LOD
NGTCI	<LOD	<LOD
NSCI	<LOD	<LOD
NTCI	<LOD	<LOD
NGTCI1	<LOD	<LOD
NGTCE	<LOD	<LOD
NOCC1	<LOD	<LOD
NOCC2	<LOD	<LOD
**Walnuts**		
OGBE	<LOD	<LOD
OGBE1	<LOD	<LOD
OGCE	<LOD	<LOD
OGCE1	<LOD	<LOD
OGCE2	<LOD	<LOD
OGCE3	<LOD	<LOD
OGCE4	<LOD	<LOD
OGCE5	<LOD	<LOD
OGCE6	<LOD	<LOD
OGCE7	<LOD	<LOD
OGCE8	<LOD	<LOD
OGCI1	<LOD	<LOD

https://doi.org/10.3390/toxins18010012

Table S11. (Continued)

**Samples**	**[AFB1] µg/kg ± SD**	**[TOTAL AFs] µg/kg ± SD**
**Walnuts**		
OGCI2	<LOD	<LOD
OGCI3	<LOD	<LOD
OGCI4	<LOD	<LOD
OGCI5	<LOD	<LOD
OGCI6	<LOD	<LOD
OGCI7	<LOD	<LOD
OS1BE	<LOD	<LOD
OSBE1	<LOD	<LOD
OSCE	<LOD	<LOD
OSCE1	<LOD	<LOD
OSCE2	<LOD	<LOD
NOC1	<LOD	<LOD

https://doi.org/10.3390/toxins18010012

Table S12. List of samples divided according to the matrix.

Samples	Characteristics	Productions	Origin
**Peanuts**			
ATCE	Roasted peanuts in shell	Conventional	Israel
ATCE1	Roasted peanuts in shell	Conventional	Egypt
ATCE2	Roasted peanuts in shell	Conventional	USA (California)
ATCE3	Roasted peanuts in shell	Conventional	Israel
ATCE4	Roasted peanuts in shell	Conventional	Israel
**Almonds**			
MPCE	Peeled almonds	Conventional	USA (California)
MS2CI	Shelled almonds	Conventional	Italy
MSCE1	Shelled almonds	Conventional	Chile
MSCE2	Shelled almonds	Conventional	USA (California)
MSCE3	Shelled almonds	Conventional	USA (California)
MGCI1	Almonds in shell	Conventional	Italy
**Hazelnuts**			
NGCI	Hazelnuts in shell	Conventional	Italy
NGTCI	Toasted hazelnuts in shell	Conventional	Italy
NSCI	Shelled hazelnuts	Conventional	Italy
NTCI	Toasted and peeled hazelnuts	Conventional	Italy
NGTCI1	Toasted hazelnuts in shell	Conventional	Italy
NGTCE	Toasted hazelnuts in shell	Conventional	USA (California)
NOCC1	Hazelnuts in shell	Conventional	Italy
NOCC2	Hazelnuts in shell	Conventional	Italy
NGCI	Hazelnuts in shell	Conventional	Italy
**Walnuts**			
OGBE	Walnuts in shell	Organic	France
OGBE1	Walnuts in shell	Organic	France
OGCE	Walnuts in shell	Conventional	Australia
OGCE1	Walnuts in shell	Conventional	France
OGCE2	Walnuts in shell	Conventional	France
OGCE3	Walnuts in shell	Conventional	France
OGCE4	Walnuts in shell	Conventional	Australia
OGCE5	Walnuts in shell	Conventional	Chile
OGCE6	Walnuts in shell	Conventional	France
OGCE7	Walnuts in shell	Conventional	USA (California)
OGCE8	Walnuts in shell	Conventional	USA (California)
OGCI1	Walnuts in shell	Conventional	Italy
OGCI2	Walnuts in shell	Conventional	Italy
OGCI3	Walnuts in shell	Conventional	Italy
OGCI4	Walnuts in shell	Conventional	Italy

https://doi.org/10.3390/toxins18010012

Table S12. (Continued)

Samples	Characteristics	Productions	Origin
**Walnuts**			
OGCI5	Walnuts in shell	Conventional	Italy
OGCI6	Walnuts in shell	Conventional	Italy
OGCI7	Walnuts in shell	Conventional	Italy
OS1BE	Shelled walnuts	Organic	Moldavia
OSBE1	Shelled walnuts	Organic	Argentina
OSCE	Shelled walnuts	Conventional	Moldavia
OSCE1	Shelled walnuts	Conventional	Chile
OSCE2	Shelled walnuts	Conventional	Chile
NOC1	Walnuts in shell	Conventional	Italy

Table S13. Precursor and product ions with collision energy (eV) for the aflatoxins evaluated by mass spectrometric analyses. The product ions chosen for quantification are shown in bold.

Compounds	Abbreviation	Formula	Retention time (minutes)	Precursor Ion	Product Ions (collision energy, eV)
Aflatoxin B_1	AFB1	$C_{17}H_{12}O_6$	5.38	313 (+)	241(34)/**285(20)**
Aflatoxin B_2	AFB2	$C_{17}H_{14}O_6$	4.06	315 (+)	243(38)/**287(22)**
Aflatoxin G_1	AFG1	$C_{17}H_{12}O_7$	4.20	329 (+)	243(22)/**311(18)**
Aflatoxin G_2	AFG2	$C_{17}H_{14}O_7$	3.24	331 (+)	245(26)/**313(23)**

https://doi.org/10.3390/toxins18010012

Table S14. Recovery, LOD, LOQ, and R^2 for each matrix. SD: standard deviation.

Samples	Analyte	Recovery [%] ± SD	LOD [ng/g]	LOQ [ng/g]	R^2
Peanuts	AFB1	92.2±8.3	3.44	11.46	0.9997
	AFB2	100.2±2.1	6.90	23.01	0.9997
	AFG1	109.0±32.1	23.61	78.71	0.9991
	AFG2	89.2±6.5	2.82	9.40	0.9987
Almonds	AFB1	86.5±5.4	2.11	7.02	0.9993
	AFB2	99.2±23.2	13.70	45.67	0.9996
	AFG1	84.7±14.2	6.01	20.03	0.9982
	AFG2	78.3±8.9	5.25	17.49	0.9988
Hazelnuts	AFB1	82.5±2.4	3.38	11.27	0.9991
	AFB2	94.9±10.9	17.48	58.27	0.9994
	AFG1	76.9±5.1	4.06	13.54	0.9995
	AFG2	97.5±26.3	4.50	15.00	0.9997
Walnuts	AFB1	80.1±9.8	6.96	23.21	0.9928
	AFB2	93.4±25.4	85.40	284.6	0.9972
	AFG1	81.8±17.1	52.34	174.5	0.9889
	AFG2	75.2±15.5	21.16	70.54	0.9996

https://doi.org/10.3390/toxins18010012
